# Ailanthone synergizes with PARP1 inhibitor in tumour growth inhibition through crosstalk of DNA repair pathways in gastric cancer

**DOI:** 10.1111/jcmm.18033

**Published:** 2023-11-27

**Authors:** Chunming Wang, Tingzhuang Yi, Xiangde Li, Jiarui Cui, Biqi Li, Yankai Qin, Shixiong Tang, Jianfeng Zhang

**Affiliations:** ^1^ Department of General Surgery The Second Affiliated Hospital of Guangxi Medical University Nanning Guangxi China; ^2^ Department of Oncology Affiliated Hospital of YouJiang Medical University For Nationalities Baise China; ^3^ Department of Radiotherapy The Second Affiliated Hospital of Guangxi Medical University Nanning Guangxi China; ^4^ College of Stomatology Shanghai Jiao Tong University Shanghai China; ^5^ Department of Pathology The Second Affiliated Hospital of Guangxi Medical University Nanning Guangxi China; ^6^ Department of Emergency The Second Affiliated Hospital of Guangxi Medical University Nanning Guangxi China

**Keywords:** ailanthone, gastric cancer, PARP1, synergizes, tumour growth inhibition

## Abstract

In our previous research, we proved that ailanthone (AIL) inhibits the growth of gastric cancer (GC) cells and causes apoptosis by inhibiting P23. However, we still find some GC organoids are insensitive to AIL. We have done some sequencing analysis and found that the insensitive strains are highly expressed in PARP1. In this study, we investigated whether AIL can enhance the anti‐tumour effect of PARPi in GC. CCK8 and spheroid colony formation assay were used to measure anti‐tumour effects. SynergyFinder software was used to calculate the synergy score of the drug combination and flow cytometry was used to detect apoptosis. Western blot, IHC, IF tests were used to measure protein expression. Finally, nude mouse xenograft models were used to verify the in vitro mechanisms. High expression of PARP1 was found to be the cause of drug insensitivity. When AIL is paired with a PARP1 inhibitor, olaparib (OLP), drug sensitivity improves. We discovered that this combination functions by blocking off HSP90‐BRCA1 interaction and inhibiting the activity of PARP1, thus in turn inhibiting the homologous recombination deficiency and base excision repair pathway to finally achieve synthetic lethality through increased sensitivity. Moreover, P23 can regulate BRCA1 in GC in vitro. This study proves that the inhibitory effect of AIL on BRCA1 allowed even cancer cells with normal BRCA1 function to be sensitive to PARP inhibitors when it is simultaneously administered with OLP. The results greatly expanded the scope of the application of PARPi.

## 1 BACKGROUND

Gastric cancer (GC) is ranked among the most common malignancies, and it maintains the third place in the cause of cancer‐related death.^1^ It is mainly found in northeastern Asians, with China leading the pack as the most afflicted country.^2^ Surgery is considered to be the best option when it comes to GC treatment, albeit being limited to earlier stage patients. However, most GC patients are diagnosed later down the line, after the window for surgery has long passed. Therefore, exploring alternative treatment solutions for subspecies of GC is essential to levelling the field. In a previous study, while sifting through multiple natural plants, we found ailanthone (AIL) to be a potent anti‐GC drug.^3^ However, as more and more GC organoid systems are established, we found two out of six of them to be insensitive to AIL. Sequencing analysis found that the insensitive strains are highly expressed in PARP1, so we believe that PARP1 could be the key gene leading to insensitivity, which we later proved both in vitro and in vivo. On the contrary, the PARP1 inhibitor, olaparib (OLP), has always been used to sensitize GC with high expression of PARP1. This important discovery showed that AIL could still work its magic on BRCA1 when AIL is used in tandem with OLP, even in GC with normal BRCA1 but highly expressing PARP. This revelation greatly expanded the usability of PARPi (PARP inhibitor).

## 2 METHODS AND MATERIALS

### 2.1 Cell culture

AGS and SGC7901 cells used in this study were ordered from Kegen Biotechnology Co., Ltd (Nanjing, China). These cell lines were cultured in RPMI‐1640 (Gibco, Grand Island, USA) supplemented with 10% fetal bovine serum in 5% CO2.

### 2.2 Human tissue and organoids

GC Tissue used for organoid culture was derived from GC patients after surgery in the Second Affiliated Hospital of Guangxi Medical University, with signed consent forms from the patients. The study was approved and supervised by the Ethics Committee of Clinical Research and Animal Experiments of the Second Affiliated Hospital of Guangxi Medical University (No. 2020‐108). This research conforms to the ethics of all animal and human tissue research. Specimens that met the standards were then screened through the Scientific Research Center of the Seventh Affiliated Hospital of Sun Yat‐sen University, and finally organoid specimens of four patients were ultimately selected in this study. Organoids were generated as follows: GC samples were placed in 50 μmL of ice‐cold PBS, minced on ice, in a gradient containing 1 mg in DMEM. 15 ml −1 Collagenase V (Sigma‐Aldrich) was fermented at 37°C for 1 h. Add ice‐cold DMEM to stop digestion, then centrifuge at 4°C (300 G, 5 min). Digestion was further performed with TrypLE (Thermo Fisher Scientific) for 5 min at 37°C and then stopped with copious amounts of DMEM. Filter the suspension with a 70 μm purification mesh, centrifuge, and resuspend the cells in the culture medium.^4^ The medium used for the establishment and cultivation of human GC organoids was as described elsewhere.^5^

### 2.3 Cell viability

In a 96‐well transparent bottom black board, 3000 cells were seeded into each well (organoids were seeded in Matrigel). Medications were added in each well according to a fivefold or 10‐fold concentration gradient. After 72 h, a luminometer (PerkinElmer Life and Analytical Sciences, Boston, MA) was used to determine the level of adenosine triphosphate (ATP) by CellTiter‐Glo Luminescent Cell Viability Assay (Promega, Madison, WI).

### 2.4 Spheroid colony formation assay

The specific method of the spherical colony formation test was as described in the Cell viability part.^5^ Human GC cells are sown in wells (500 cells/or indicated) in ultra‐low‐attachment 24‐well plates supplemented with 2 mL DMEM/F12 medium (glico) and 10 mM HEPES, recombinant human epidermal growth factor (EGF) (Invitrogen) at a concentration of 20 ng/mL, and human recombinant basic fibroblast growth factor (bFGF) (Invitrogen) at a concentration of 10 ng/mL. After 2–4 weeks, each well was inspected with a light microscope, and the spherical colonies were counted in five random fields of view.

### 2.5 Flow cytometry and FACS

The method used was the same as the standard method we published.^6^ Cellular apoptosis was tested by means of using the Annexin V‐FITC Apoptosis Detection Kit (Sigma‐Aldrich, St. Louis, MS, USA), and following the protocol provided by the manufacturer. The obtained data were evaluated using the FlowJo 10 software.

### 2.6 Biochemistry and immunohistochemical staining

Mice in the subcutaneous xenograft model were sacrificed, and their serum, kidney, liver and heart were collected by the end of the experiment to evaluate the toxicity of WA. Serum levels of aspartate aminotransferase (AST) and alanine aminotransferase (ALT) were measured. Kidney, liver and heart were stained with haematoxylin and eosin (Figure S2A–C) and then imaged under a microscope.

In the immunohistochemical staining assay, all the animal tissue samples were initially fixed in a 10% neutral buffered formalin, and later processed by dehydration, and paraffin embedding. The samples were then sectioned at 3 μm thickness. According to the protocols provided by the manufacturer, we performed IHC staining utilising these fixed and paraffin‐embedded tissues with an Immunohistochemistry Kit (Sangon Biotech, Shanghai, China). Primary antibodies were used for the IHC staining. Horseradish peroxidase‐conjugated streptavidin was later introduced for 30 min. Positive cells were counted using different fields under a microscope after DAB staining. The staining intensity score of this study was defined according to the formula as described in the literature.^5^

### 2.7 PARP1‐targeting shRNA (short hairpin RNA) and lentivirus packaging

Lentivirus packaging was completed as described in a previous study.^7^ Lipofectamine 2000 (Life Technologies, California, USA) was used for shRNA transfection according to the protocol provided by the manufacture. To establish stable P23 knocked‐down cell lines, we cloned oligonucleotides encoding short hairpin RNA (shRNA) into lentiviral vector psi‐LVRU6GP (Genecopoeia), which were named as sh‐1 (AATGAAGACATAGTCCCTTCG), sh‐2 (ATCTGCTCCATCTACTTCTGG), sh‐3(TTTATGCTTGGAATCATTTGG) respectively. A scrambled shRNA named as sh‐Ctrl was used as a negative control in this study. After being transfected with sh‐RNAs (sh‐1, sh‐2, sh‐3 or sh‐Ctrl), GC cells were later cultured in puromycin‐supplemented medium (2 μg/mL puromycin) for 4 weeks to establish stable cell lines.

### 2.8 Quantitative real‐time PCR

Trizol reagent (Accurate Biology, Changsha, China) was used to extract total RNA according to the protocols provided by the manufacturer. qRT‐PCR was performed according to the protocols provided by the manufacture using Evo M‐MLV reverse transcriptase premix and SYBR Green Premix Pro Taq HS qPCR kit (Accurate Biology). P23 PCR primer is: Forward Sequence (CTCGGAGGAAGTGATAATTTTAAGC), Reverse Sequence (GCCATGACTGTCCAGATTCTCC).

### 2.9 Western blotting

We extracted the total protein from cells on ice. Protein concentration was measured and separated with the method of SDS‐PAGE (Beyotime) at 95° for 8 min, then transferred onto PVDF membranes (Millipore, Billerica, USA). These PVDF membranes loaded with the transferred proteins were soaked in 5% BSA solution for at least 1 h, they are then immediately incubated at 4°C overnight with different primary antibodies. On the second day, the PVDF membranes were washed with TBST solution three times and again with secondary antibodies at room temperature for a minimum of 1 h. After a 30‐min wash with TBST solution, the antibody‐conjugated protein bands on the PVDF membranes were visualized under an imaging system (Bio‐Rad ChemiDoc MP) with a BeyoECL Plus Kit (Beyotime Biotechnology). Antibodies used included γH2AX (1:100, Abcam, ab229914), Caspase3 (1:200, Proteintech, 19677‐1‐AP), RAD51(1:100, Abcam, ab133534) PARP‐1 antibody (F‐2) (1: 500, Santa Cruz, sc‐8007), and XRCC1 (1:100, Abcam, ab134056). BRCA1 (1:100, Proteintech, 22362‐1‐AP). P23 (1:50, Santa Cruz, sc‐136021). HSP90 (1:500, Cell Signaling Technology, #4874). Histone H3 (1:500, Cell Signaling Technology, #4499). Histone H3 (1:500, Cell Signaling Technology, #4499). beta tubulin (1:10000, Proteintech, 10094‐1‐AP). GAPDH (1:10000, Proteintech, 60004‐1‐Ig).

### 2.10 Immunofluorescent staining

Immunofluorescence staining of organoids, tumour tissues and cell lines was performed using the method we previously published.^6^ Fluorescence staining was imaged on a Zeiss LSM780 confocal microscope. Antibodies used included γH2AX (1:100, Abcam, ab229914), Caspase3 (1:200, Proteintech, 19,677‐1‐AP), RAD51(1:100, Abcam, ab133534) PARP‐1 antibody (F‐2) (1: 500, Santa Cruz, sc‐8007), and XRCC1 (1:100, Abcam, ab134056).

### 2.11 Immunoprecipitation

To evaluate the interaction of WA on HSP90 and BRCA1, an immunoprecipitation assay was performed. After cell lysis using RIPA Lysis Buffer (Beyotime, Shanghai, China), and centrifugation, the supernatant was collected. Protein concentration was measured at 2000 μg of protein lysate by BCA Protein Assay Kit (Beyotime). Then antibody was added to the lysates with protein A beads (Thermo Fisher Scientific, CAT#20424) and left to incubate overnight. The beads were then Western blotted.

### 2.12 Animal experiments

In vivo experiments had been performed according to the Institutional Animal Care and Use Committee (IACUC). It was approved by the clinical scientific research and animal experiment ethics committee of the Second Affiliated Hospital of Guangxi Medical University ([2021] No. 017). The experiment was conducted in the Ruiye Animal Model Center. Balb/c nude mice (female, 8 weeks old, 19–21 g in mass) were purchased from GemPharmatech Experimental Animal Co., Ltd (Nanjing, Jiangsu) and raised in an SPF environment. PDX method was as mentioned before.^8^ When the tumour volume reached about 150 mm^3^, they were randomly divided into three groups and different reagents were given by intraperitoneal injection (IP). The tumour volume and body mass were measured every 3 days. The calculation formula of tumour volume is as follows: V = Πab^2^/8 (where V is the tumour volume, ‘a’ is the largest tumour diameter, and ‘b’ is the smallest tumour diameter). Nude mice were sacrificed on day 30, and the tumours were taken for measurement and weighing.

To establish the in vivo model, PARP1 gene knock‐down stable SGC7901 GC cells (2 × 10^6^ cells/mice) or shNC control cells (2 × 10^6^ cells/mice) were injected subcutaneously. Seven weeks after the injection, all these mice were sacrificed and subcutaneous tumours were removed and examined by IHC and HE staining. The dosage of AIL and OLP was according to the paper.^3^, ^4^

AIL dose was 100 mg kg^−1^, OLP dose was 20 mg kg^−1^, combined group dose was AIL 50 mg kg^−1^ and 25 mg kg^−1^ of OLP twice per week, each treatment lasting for the period of 4 weeks.

### 2.13 RNA sequencing and Screening of differentially expressed genes (DEGs)

To investigate the molecular mechanism of action, RNA sequencing was used to examine differential gene expression. GC1, GC2, GC3, GC5 and GC4, GC6 organoids were, respectively. Nanodrop 2000 was used to detect total RNA. Hybridisation was performed by Origingene Bio‐pharm Technology (Shanghai, China). And RNA sequencing was as described in the literature.^9^ The data set was normalized using the expectation–maximisation algorithm available in RNA‐Seq. In this study, the analysis of the DEGs was as described in the literature.^9^

### 2.14 Statistical analysis

All of the data were expressed as means ± standard deviation, and *p* < 0.05 was considered statistically significant. The tumour volume was determined using repeated measurements of the general linear model and log‐rank test with SPSS 18.0. Other statistical analyses used in this research include one‐way analysis of variance (anova) and the Student's *t*‐test.

## 3 RESULTS

### 3.1 PARP1 is the key gene inhibiting sensitivity to AIL

**FIGURE 1:**
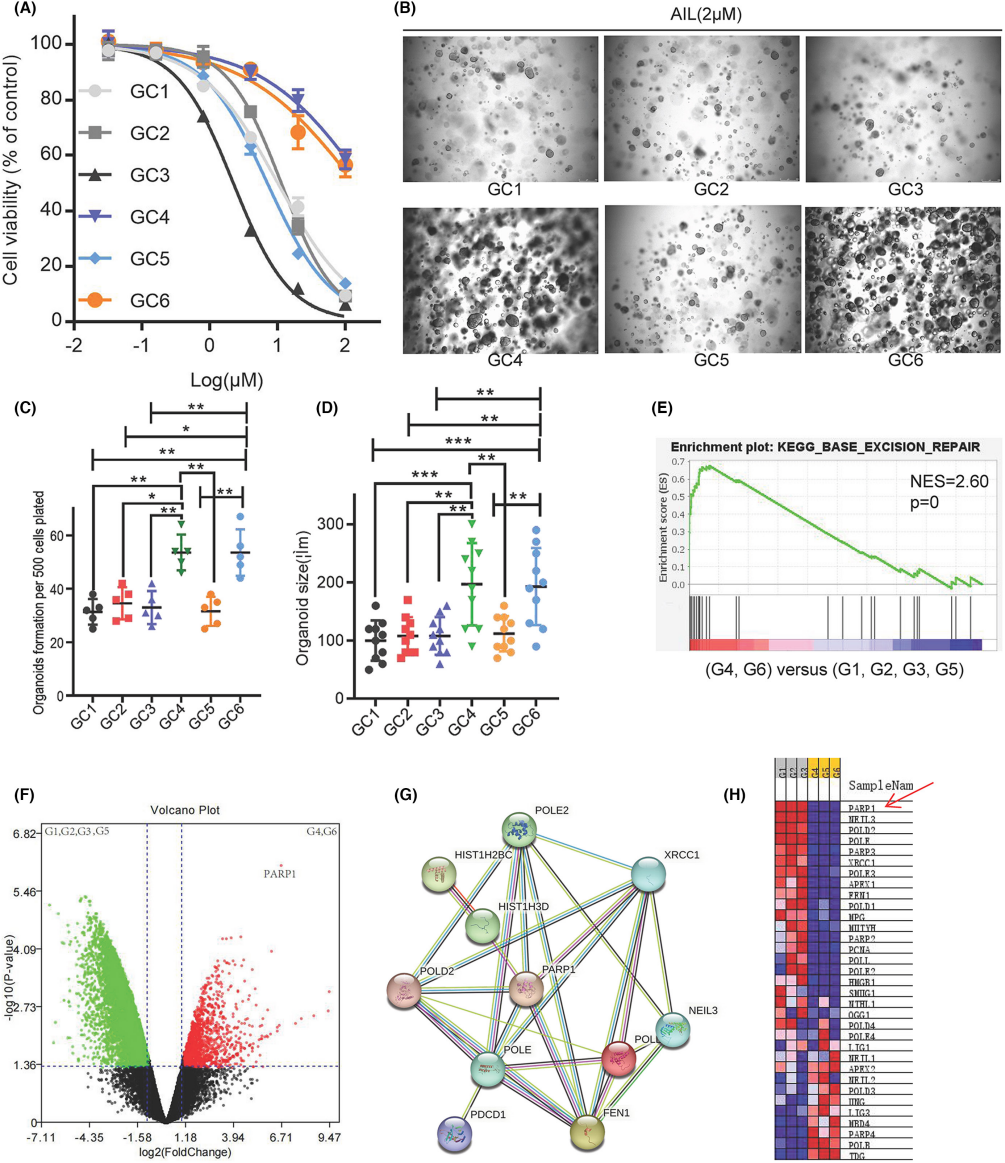
PARP1 is the key gene leading to insensitivity to AIL. The AIL used for drug screening in six GC organoids. After 72 h, cell viability was analysed via CCK8. Data were derived from experiments conducted in triplicate. (B) Representative images of GC organoids treated with 2 μM AIL for 14 days. The number of cell clusters (C) and size of clusters (D) were counted. Data were derived from experiments conducted in triplicate. (E) Analysis of enrichment of mRNA differential expression of GC1, GC2, GC3 and GC5 tumours against GC4 and GC6 tumours. (F)Differential gene expression analysis of the transcriptome sequencing. (G) STRING database protein interaction network diagram of mRNA differential expression in GC1, GC2, GC3 and GC5 tumours compared to GC4 and GC6 tumours. Edges represent protein–protein associations. Cambridge blue, curated databases; Violet, experimentally determined; Green, gene neighbourhood; Red, gene fusions; blue, gene co‐occurrence; Reseda, text mining; black, co‐expression; Lilac, protein homology. (H) Heatmap of mRNA differential expression of GC1, GC2, GC3 and GC5 tumours against GC4 and GC6 tumours. Red represents High and blue represents Low. The abscissa represents the gene name.

The three GC organoid models we previously established for drug discovery in mainstream medicinal plants found that AIL extracted from the plant *Ailanthus altissima* exhibits a great inhibitory effect on GC. However, as we continuously establish more models, two strains (GC4 and GC6) developed from GC patients with poor chemotherapy results showed insensitivity to AIL as well. Cell viability tests among the 6 strains showed that GC2 (IC_50_ = 190.3 mM) and GC5 (IC_50_ = 138.1 mM) are more resistant to AIL than the other 4 (IC_50_ = 2.2–10.1 mM) (Figure 1). Spheroid formation assay also revealed that AIL was ineffective at suppressing the number and volume of GC2 and GC5 strains (Figure 1B–D). To seek out the possible key gene that caused this insensitivity, we ran an mRNA sequencing on all 6 GC organoids through mapping a protein–protein interaction network. Differentially expressed genes and fold change are shown in Figure 1F. Compared to sensitive strains, the enriched pathway of the insensitive one's tops pathway includes the base excision repair pathway (Figure 1E). Finally, we used STRING to search for the core gene, which then revealed PARP1 as the candidate gene for drug resistance (Figure 1G,H).

### 3.2 AIL synergistically increases cell toxicity to GC cells when used with OLP

**FIGURE 2:**
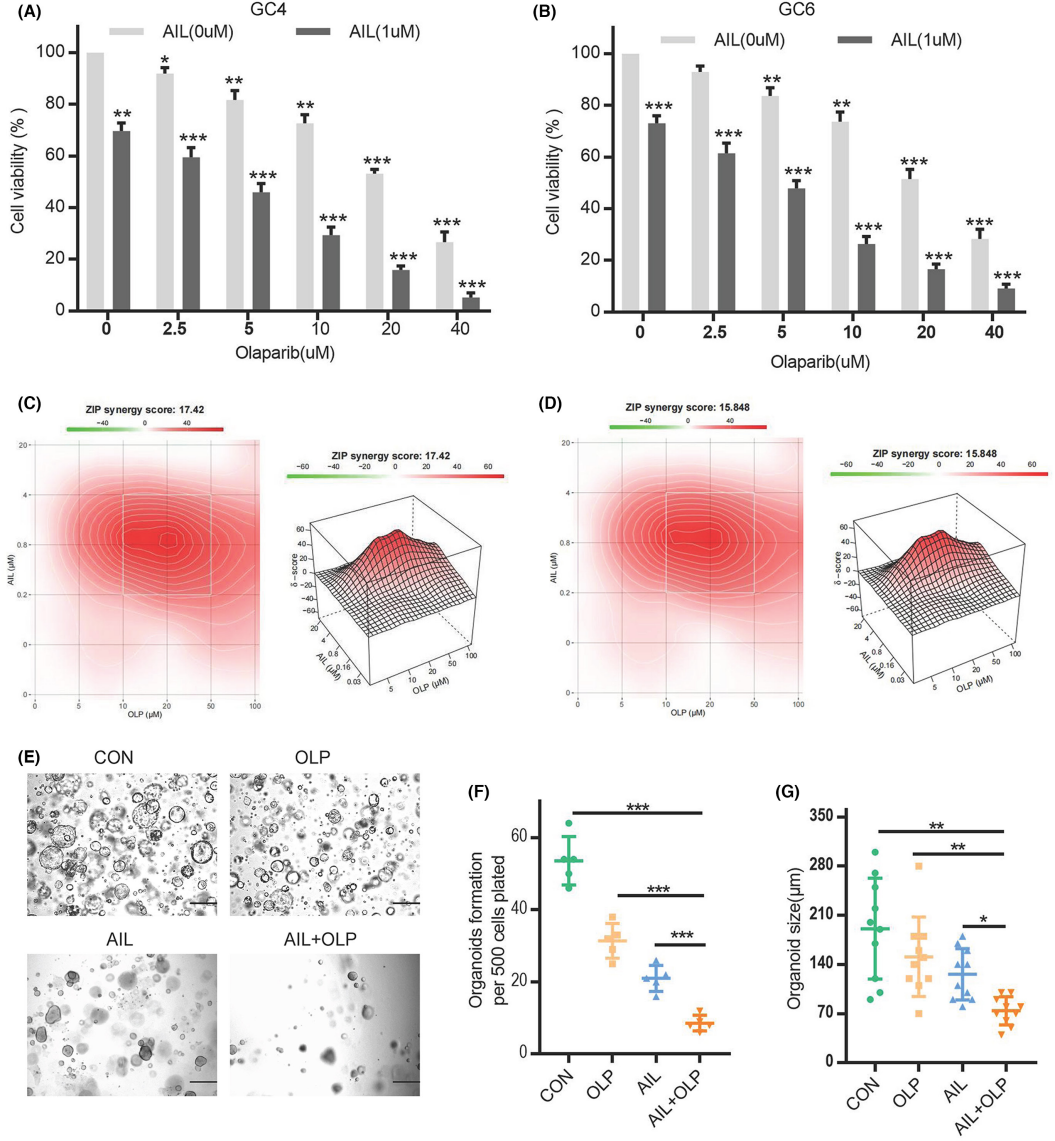
AIL with Olaparib can increase cell toxicity to GC cells. GC4 organoids were treated with a blank control group, OLP, AIL, and OLP + AIL group. At 72 h after treatment, the cell viability was determined by CCK8 assay. (B) GC6 organoids were treated with a blank control group, OLP, AIL, and OLP + AIL group. At 72 h after treatment, the cell viability was determined by CCK8 assay. (C, D) ZIP synergy plots of combined treatments of OLP and different AIL drugs on GC4 and GC6 organoids. The synergy score was calculated using SynergyFinder software. Less than −10: the interaction between two drugs is likely to be antagonistic; from −10 to 10: the interaction between two drugs is likely to be additive; larger than 10: the interaction between two drugs is likely to be synergistic. (E–G) Representative images of GC4 organoids treated with a blank control group, OLP, AIL, and OLP + AIL group for 14 days. The number of cell clusters (F) and size of clusters (G) were counted. Data were derived from experiments conducted in triplicate.

In an additional test, we have shown that the combination of AIL and OLP which is a PARP1 inhibitor can successfully impede the growth of AIL insensitive strains, namely GC4 and GC6 (Figure 2A,B). And we also proving their synergy, and found the combine of the AIL and PARP inhibitor can have significant synergy (Figure 2C,D). The compound drug (AIL) also significantly suppresses the viability and proliferation of AIL resistant strains GC2 and GC5. We have indubitably proven that this mixed drug can substantially inhibit the proliferation of GC4, as shown through the results from cell viability in organoid spheroidization tests (Figure 2E,F), and its volume (Figure 2E–G). And we also try the AGS and SGC7901, show AIL synergistically increases cell toxicity to GC cells when used with OLP (Figure S1A–C).

### 3.3 AIL with OLP induces apoptosis through aggravating DNA damage in GC cells

**FIGURE 3:**
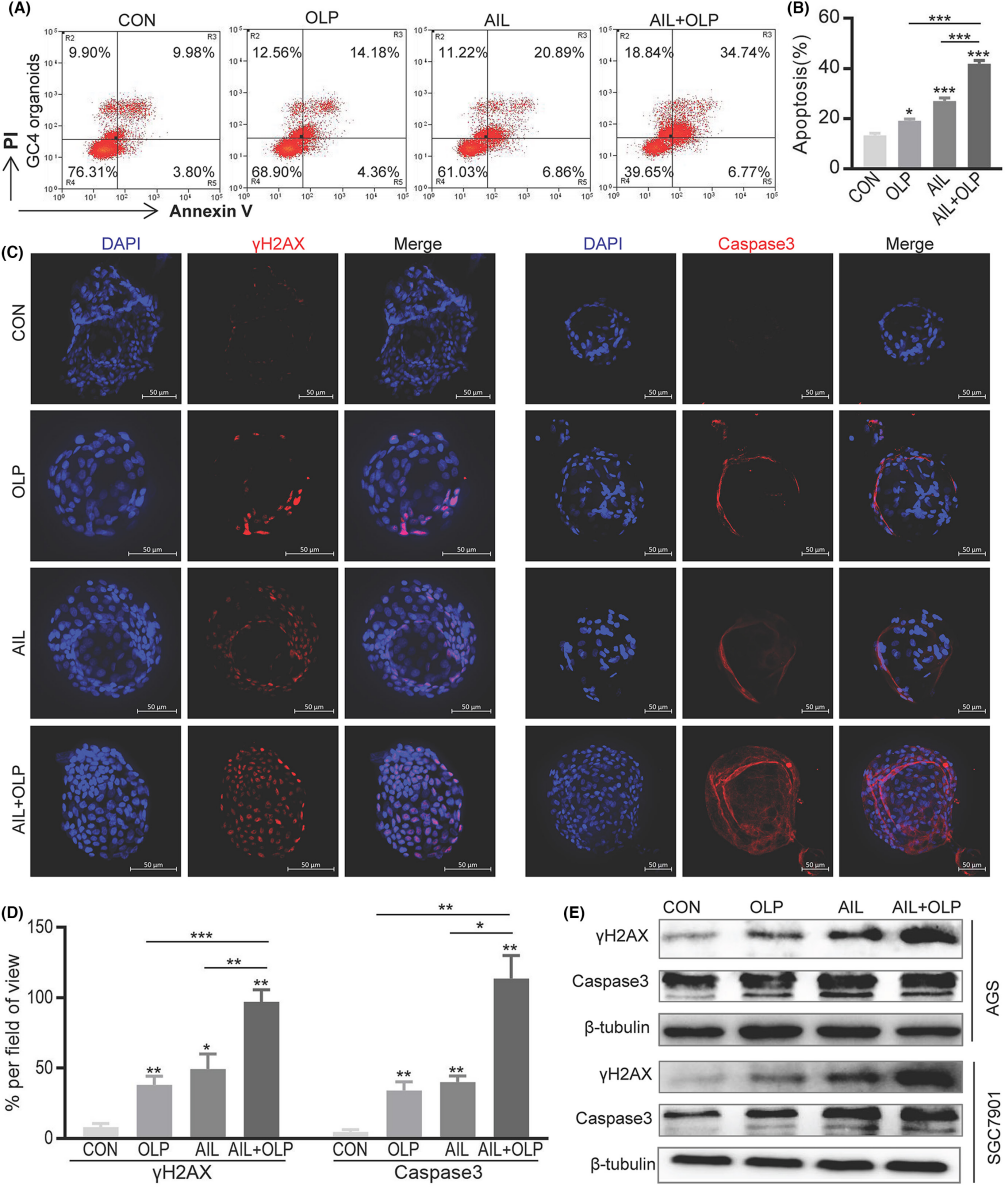
AIL with Olaparib led to apoptosis via aggravating DNA damage in GC cells. Three GC4 organoids were treated with OLP and AIL alone or in combination with the indicated concentrations for 48 h, followed by digestion into single cells and analysis of apoptosis through flow cytometry analysis with FITC‐Annexin V and PI staining. OLP: 5 nM, AIL: 100 nM. (B) Data were derived from experiments conducted in triplicate in (C). Representative images of γH2AX and Caspase3 stained by IF staining of GC4 organoids treated with a blank control group, OLP, AIL, and OLP + AIL group. The red stains indicate γH2AX and Caspase3 positive. (D) Statistical analysis of γH2AX and Caspase3 + cells. (E) Comparison of γH2AX and Caspase3 expression in a blank control group, OLP, AIL, and OLP + AIL group in AGS, and SGC7901 cells for 24 h.

First, our research into the effects of AIL with OLP on the apoptosis of resistant organoids demonstrated that the AIL+OLP group has greater rate of apoptosis when compared to single‐drug AIL or OLP, and control (Figure 3A,B). We have also replicated similar results when we did immunofluorescence (Figure 3C,D) and western blot (Figure 3E) on Caspase3, which is an apoptotic marker in organoids. Since PARP1 is an important gene in the BER pathway of DNA repair,^10^ and AIL suppresses cancer by inhibiting DNA repair.^3^ we wanted to establish the changes to DNA when AIL is used with OLP on AIL insensitive GC organoids. In the immunofluorescent test of the organoid DNA damage protein γH2AX, we found that the AIL+OLP group holds an advantage in DNA damage over the other single‐drug or control groups (Figure 3C,D). Western blot results also supported this finding (Figure 3E).

### 3.4 AIL with OLP blocks HSP90‐BRCA1 and PARP1 activity

**FIGURE 4:**
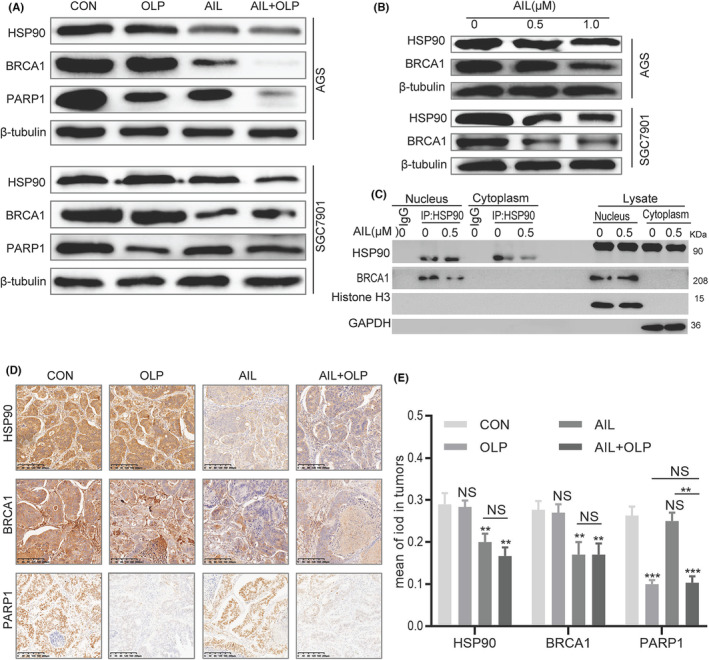
AIL with OLP blocks HSP90‐BRCA1 and PARP1 activity. The protein expression of HSP90, BRCA1, PARP1 in two GC cell lines after treated with a blank control group, OLP, AIL and OLP + AIL group. (B) The protein expression of HSP90, BRCA1 in two GC cell lines after being treated with a specified concentration of AIL for 24 h. (C) Pull‐down of selected proteins with HSP90 in the nucleus and cytoplasm of GC cells treated with AIL for 24 h, and then the IP fractions were immunoblotted with HSP90, BRCA1 and GAPDH. (D) Representative images of HSP90, BRCA1 and PARP1 in PDX tumour tissue as detected via immunohistochemistry. Scale bars, 200 μm. (E) Data were derived from experiments conducted in triplicate in (D). The PDX tumour tissues were compared with the control group, *p < 0.05, **p < 0.01, ***p < 0.001.

Our results above indicated that OLP can sensitize AIL insensitive GC organoids, thus drastically increasing DNA damage, forming synthetic lethality when used with OLP as AIL inhibited the homologous recombination (HR) pathway. Furthermore, as an essential gene for single‐strand DNA repair, BRCA1 had to be malfunctioning before PARP1 activity could be inhibited.^10^ To confirm that AIL with OLP functions through inhibiting PARP1 and BRCA1, we explored the changes in HSP90, BRCA1 and PARP1 protein levels via western blotting. It turned out that OLP was impotent on BRCA1, while AIL was ineffective on PARP1 but effective on BRCA1. Meanwhile, AIL with OLP has a significant effect on PARP1 (Figure 4A). Since BRCA1 is the client protein of HSP90.^11^, ^12^ we further western blotted the changes in HSP90 and BRCA1 proteins against different concentrations of AIL, which exposed their concentration dependence (Figure 4B). Lastly, Co‐Immunoprecipitation tests revealed that AIL inhibits the HSP90‐BRCA1 protein interactions as well. Hence, our leading hypothesis is that AIL inhibits HSP90‐BRCA1, and OLP suppress the activities of PARP1 (Figure 4C). At the meantime, we also validated the relationship the finding of the result of in‐vitro. According to the PDX IHC of BRCA1, PARP1 and HSP90, we have the same conclusion of in vitro data (Figure 4D,E).

### 3.5 AIL suppresses BRCA1 by downregulating P23 and thereby inhibits the homologous recombination pathway in GC

**FIGURE 5:**
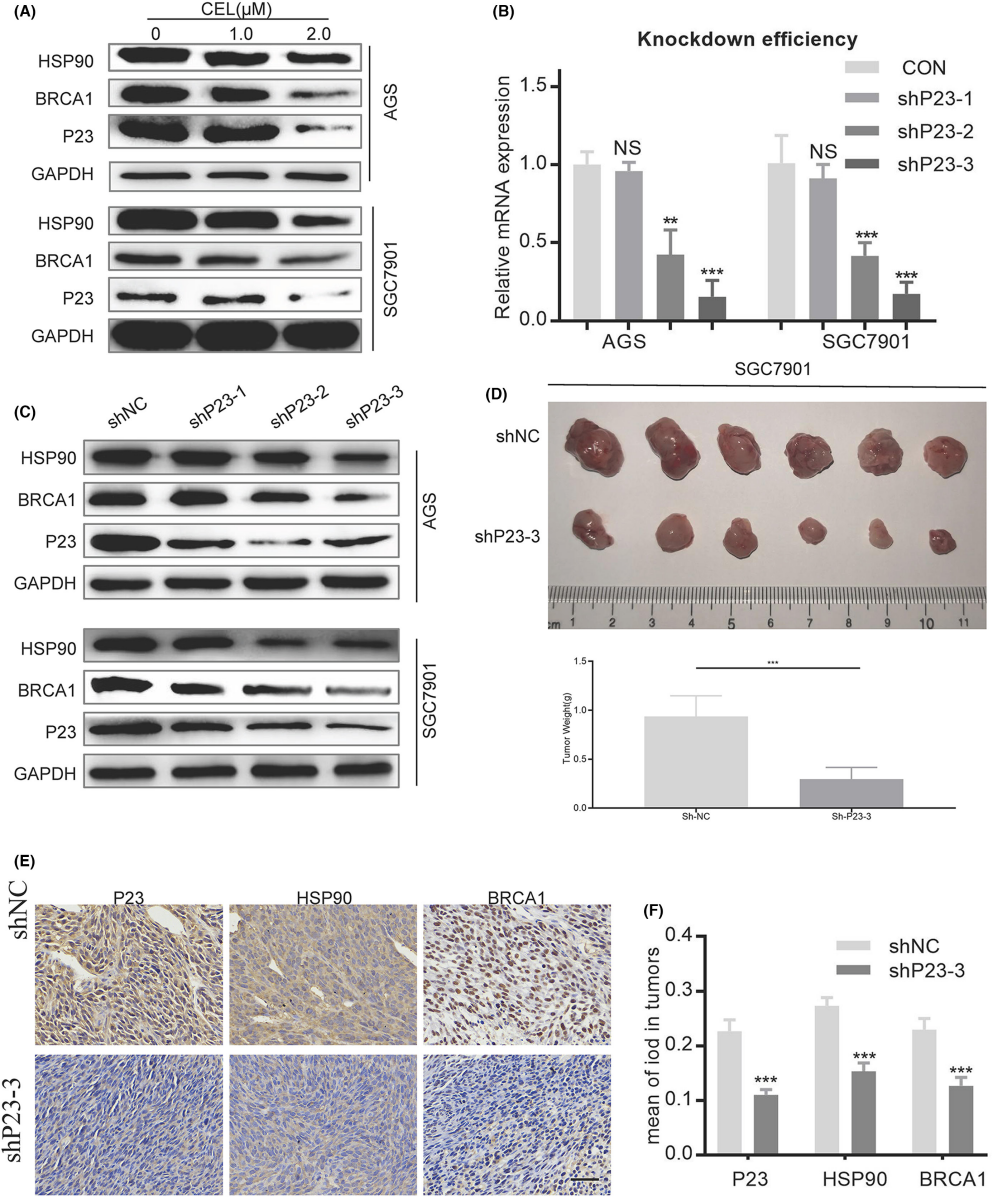
AIL inhibits BRCA1 through downregulating P23 and inevitably suppress the HR pathway of GC. Western blotting was performed to detect the expression of P23, HSP90 and BRCA1 in AGS and SGC7901 cells after treatment with a specified concentration of CEL (P23 inhibitor) for 24 h. (B) The mRNA expression of P23 in two GC cell lines (AGS and SGC7901) after being treated with P23 shRNA lentivirus. (C) The protein expression of P23, HSP90 and BRCA1 in two GC cell lines after being treated with shP23‐1, shP23‐2 and shP23‐3. (D‐F) BALB/C nude mice are implanted with shNC or shP23‐3 GC cells subcutaneously. (D) The images of subcutaneous tumour nodules formed in two groups of mice. (E) Representative images of P23, HSP90 and BRCA1 in CDX tumour tissue as detected via immunohistochemistry. Scale bars, 50 μm. (F) Data were derived from experiments conducted in triplicate in (E).

Our previous project concluded that AIL can downregulate the accessory chaperone of HSP90, P23.^3^ Since BRCA1 is a client protein of HSP90, we speculate that AIL inhibits BRCA1 by downregulating P23 in GC. However, there are no relevant studies on the regulatory effect of P23 on BRCA1. To arrive at a satisfying conclusion, we applied the P23 inhibitor, celastrol (CEL), to observe the changes in the protein levels of HSP90 and BRCA1 in GC. Expectedly, CEL can effectively suppress BRCA1 (Figure 5A). A confirmatory experiment was conducted, by using shRNA to knockout P23 in 2 efficient GC cell lines, namely AGS and SGC7901 (Figure 5B). Western blot revealed reduced BRCA1 protein expression (Figure 5C). We also conducted in vivo experiments (Figure 5C–E) with immunohistochemistry results supporting the same conclusion (Figure 5D: P23 knockout SGC7901 subcutaneous GC xenograft).

### 3.6 AIL with OLP aggravates DNA damage through the inhibition of BER and HR repair pathways

**FIGURE 6:**
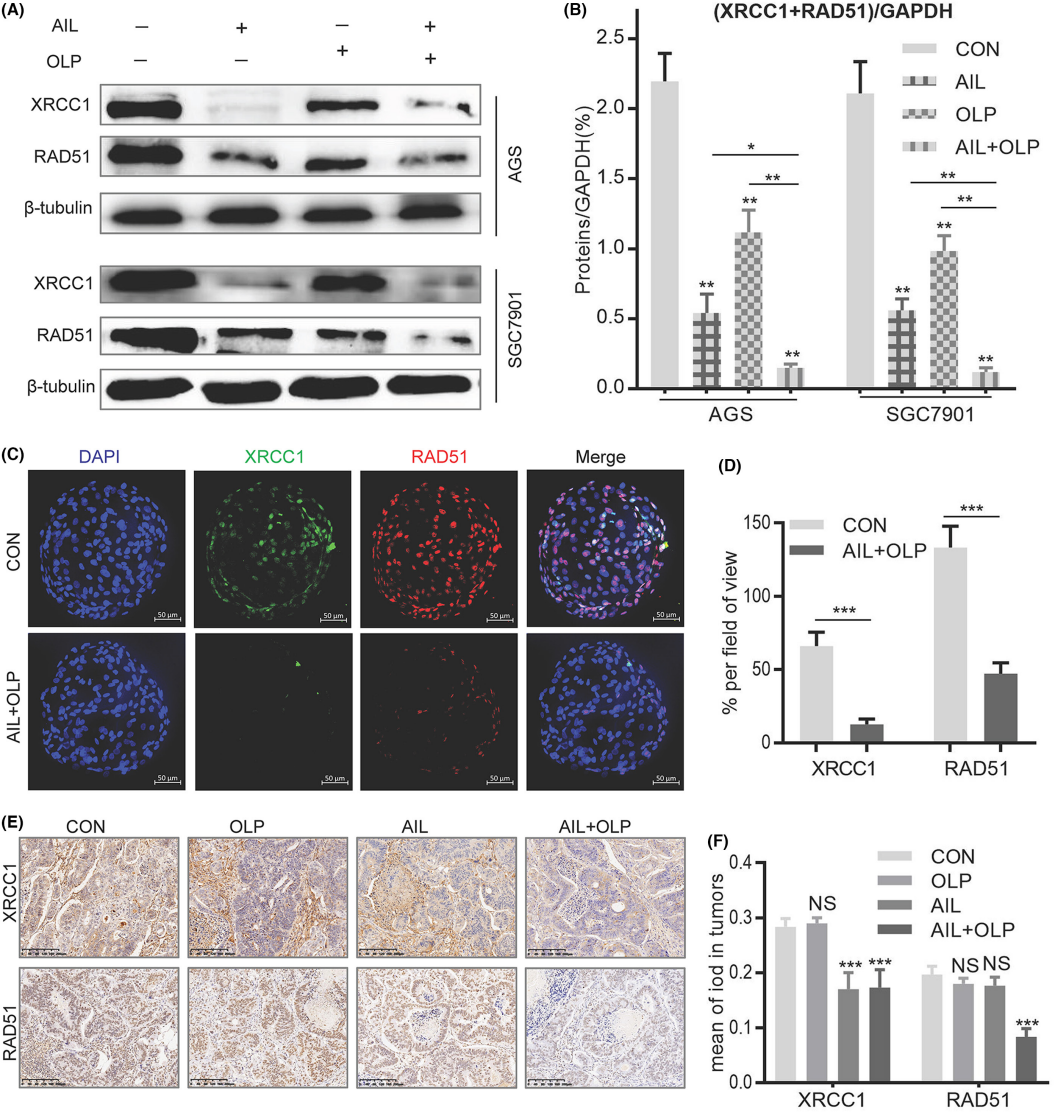
AIL with OLP aggravates DNA damage through inhibiting BER and HR repair pathway. Verification by western blot on the effects of OLP, AIL, OLP + AIL on XRCC1, RAD51 expression in two GC cell lines. (B) Data are expressed as mean ± SD from three independent experiments. (C) The colocalization of XRCC1 and RAD51 was demonstrated with immunofluorescence in GC4 organoids treated with DMSO and AIL (1 μM) + OLP (50 μM) for 24 h. Scale bars, 50 μm. (D) Statistical analysis of data in (C). (E) Representative images of XRCC1 and RAD51 stained by IHC staining after tumorigenesis of BALB/C NUDE mice treated with DMSO, OLP, AIL, OLP + AIL group of GC4. The brown stains indicate XRCC1 and RAD51 positive. The scale represents 200 μm. (F) Data were derived from experiments conducted in triplicate in (E).

Homologous recombination repair deficiency (HRD) can only be achieved through suppressing the BRCA1 function, which is the key gene in the HR pathway. HRD leads to effective HR impairment, increasing double‐strand DNA damage, finally resulting in cancer cell apoptosis.^13^ Hence, BRCA1 function impairment is the precondition to synthetic lethality. To evaluate the potential of AIL with OLP in suppressing BER and HR repair pathways, we measured the protein changes in XRCC1 and RAD51 among the control and experimental groups. Results show that AIL was effective at suppressing XRCC1, a single‐strand DNA damage repair marker in the BER pathway, it was helpless when it comes to RAD51, the key marker in the HR repair pathway. On the contrary, OLP is ineffective at suppressing both of these markers, but when used with AIL, they are much more potent (Figure 6A). Therefore, AIL combined with OLP significantly inhibited the repair of DNA single‐strand (XRCC1) and double‐strand breaks (RAD51) in GC when compared with standalone usage (Figure 6B). In AIL resistant GC organoids, immunofluorescent tests showed that the combined drug is effective at suppressing BER and HR levels when compared to the control group (Figure 6C,D). We further explored XRCC1 and RAD51 expression levels in AIL resistant GC PDX models. The findings demonstrated that AIL+OLP are also effective in vivo (Figure 6E,F). All of the above concludes that AIL when combined with OLP functions by suppressing HSP90‐BRCA1, causing HRD which then suppresses PARP1 activity, consequently inhibiting BER and HR repair pathway and thus compounds DNA damage.

### 3.7 AIL with OLP inhibits PDX growth

**FIGURE 7:**
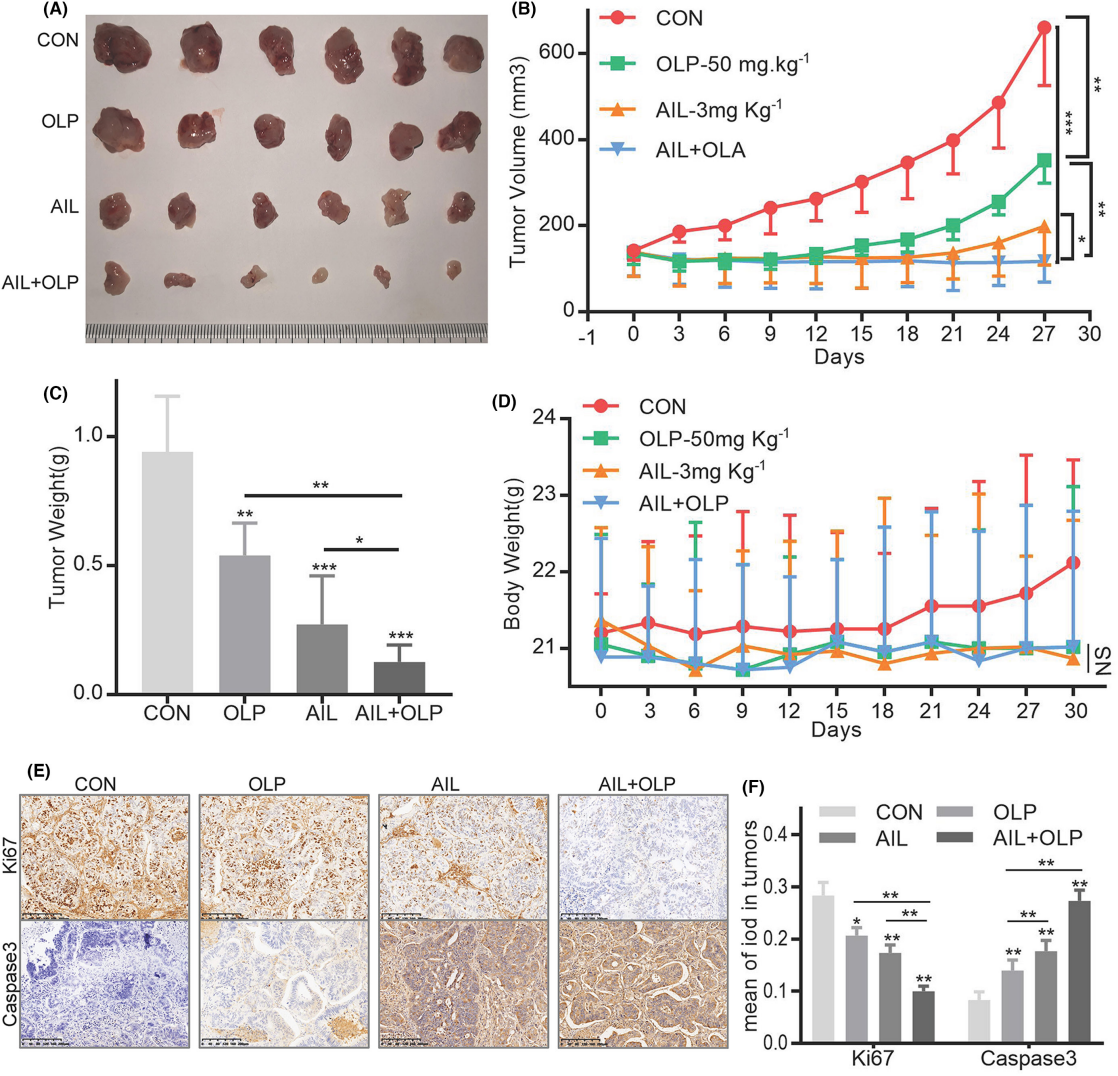
AIL with OLP inhibits in vivo growth of the PDX model. (A–D) PDX models were established with subcutaneous inoculation of nude mice with GC4 tissue. After the tumours grew to a certain volume, the mice were randomly divided into 4 groups (six mice in each group) and received daily intraperitoneal injections of DMSO, OLP, AIL, OLP + AIL for 30 days. (A) Subcutaneous tumour tissue from four experimental groups of nude mice. (B) The curve shows the average tumour volume. Error bars represent mean ± standard deviation. (C) Tumour weight statistics of subcutaneous tumour tissue from four experimental groups of nude mice. (D) Mice body mass curve. (E) Representative images of Ki67 and Caspase3 stained by IHC staining after tumorigenesis of BALB/C NUDE mice treated with DMSO, OLP, AIL, OLP + AIL group of GC4. The brown stains indicate Ki67 and Caspase3 positive. The scale represents 200 μm. (F) Data were derived from experiments conducted in triplicate in (E). *p < 0.05, **p < 0.01, ***p < 0.001.

**FIGURE 8:**
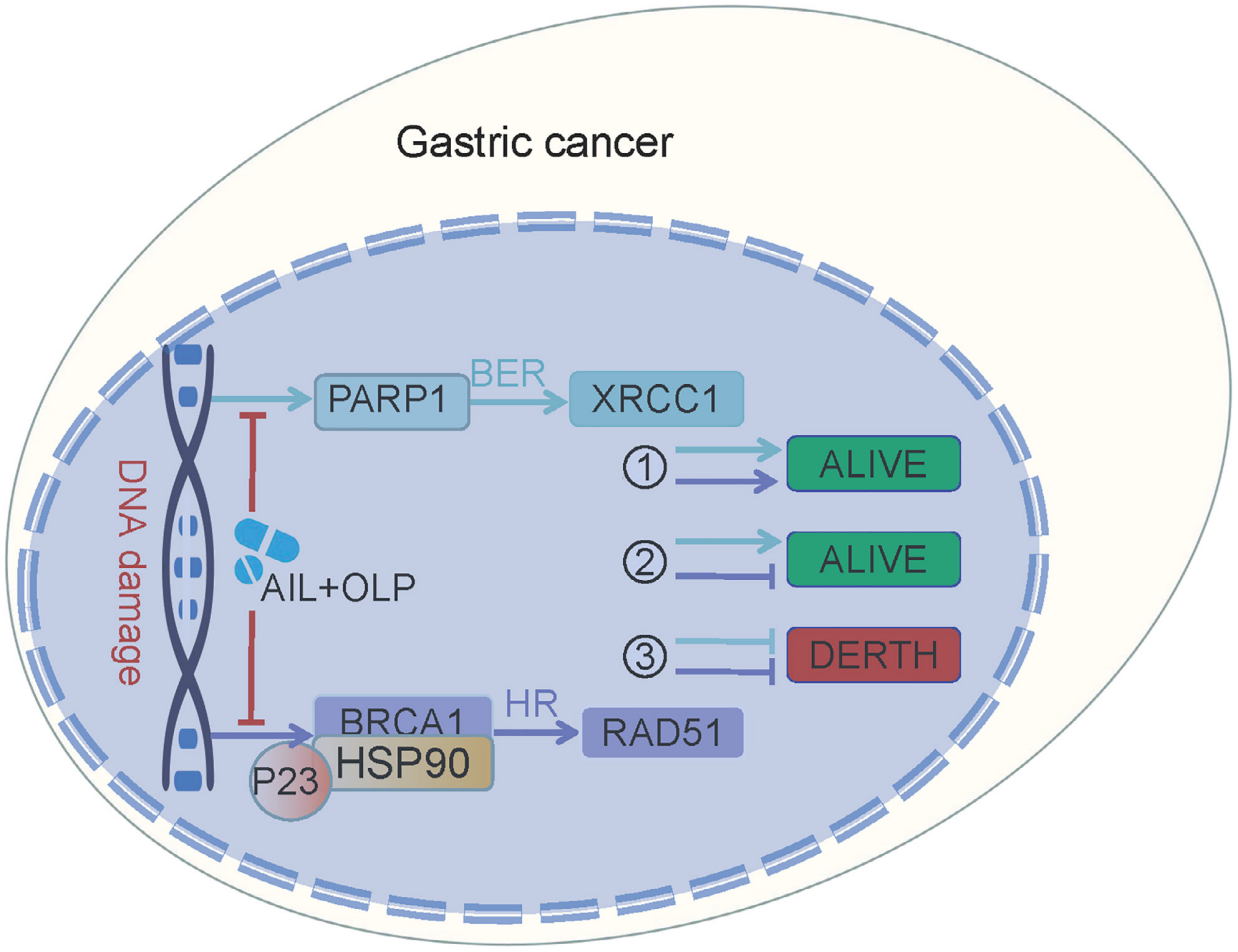
The schematic model of AIL synergizes with PARP1 inhibitor in tumour growth inhibition through crosstalk of DNA repair pathways in gastric cancer.

To evaluate the drug combination in vivo, we established a PDX with GC4 tissues. We found that while 3 mg/kg AIL or 50 mg/kg OLP are both effective at suppressing GC growth, the effects are more evident when used together (Figure 7A–C). Notably, these treatments (Con, AIL, OLP and ALP + OLP) did not significantly reduce the subjects' body mass (Figure 7D). Additionally, AIL with OLP did not affect liver and kidney function after treatment (Figure S2A–C). IHC tests of these PDX masses showed that the combined therapy significantly suppressed Caspase3 and Ki67 protein expressions while increasing the DNA damage marker γH2AX's protein expressions. All of the above proves that combined therapy is better than standalone at increasing DNA damage, inducing apoptosis of GC cells, and suppressing proliferation (Figure 8).

## 4 DISCUSSION

Gastric cancer has a generally unfavourable prognosis, and it is globally third in terms of cancer‐related death.^1^ These facts are primarily attributable to resistance to platinum‐based drugs.^14^ Ailanthone was found to serve as an anti‐cancer drug similar to platinum‐based drugs as it also suppresses DNA repair. AIL is a medicinal plant extracted from *Ailanthus altissima*. It has been used for thousands of years to treat various illnesses in many countries such as China, Japan, Thailand, etc.^15^ In recent years, AIL has been confirmed by modern scientific methods to have significant anticancer activity.^15^, ^16^, ^17^, ^18^, ^19^ In recent years, studies have shown that altosone also has an effect on DNA damage. Chen et al. showed that DNA content analysis showed that cells exposed to altosone exhibited more cell cycle arrest during the G2/M phase compared to the control group. In cancer cells, the genetic control of cell division is altered, resulting in infinite cell proliferation.^20^

Our research demonstrated AIL resistance in two out of six cultivated GC organoids. As we sought the root cause, sequencing tests brought up PARP1 as the main gene mediating the resistance to AIL. By using OLP alongside AIL, they synergistically reversed the AIL resistance in these GC strains. In vivo experiments on PDX models have similarly confirmed our initial findings. After that, we proved that AIL functions by suppressing BRCA1, which leads to homologous recombination repair deficiency (HRD) while OLP suppresses PARP1 in the BER pathway and thus the accumulating DNA double‐strand damage marker RAD51. These two circumstances brought about synthetic lethality. In an extended animal experiment, we compared BER and HR pathway markers and DNA damage markers in different experimental groups. It showed that AIL suppresses the HR pathway, while OLP deals with the BER pathway, and collectively they increased DNA damage in GC cells.

PARP1 and BRCA1 are the regulating factors in the BER and HR repair pathways respectively. As a core member in the BER pathway, the PARP1 gene encodes for adenosine diphosphate ribosyltransferase which can recognize and poly ADP‐ribosylase many different nuclear receptors.^21^ PARP1 enhances the activity of adenosine ribosyl transferase enzyme encoded in the catalytic domain, resulting in enhanced DNA repair ability.^22^ Hence, PARP is responsible for single‐stranded DNA repairs. Usually, if a single base break occurs during repair, it is considered common DNA damage that is usually harmless to cells. At this point, the cell activates BRCA for high‐fidelity repair (HR) of the double‐strand break.^23^ However, if broken bases are not repaired in time and are transcribed or copied, they will be destroyed and damage the new DNA copies; and if BRCA1 of the HR repair pathway is lost or suppressed, meaning HRD, then harmful DNA with double‐strand breaks accumulates, causing unstable DNA and eventually, cell death, and this sequence of events form the basis of synthetic lethality.^13^ These were all theories until 2014, when OLP, the first PARP inhibitor based on the synthetic lethal mechanism, was approved for the treatment of ovarian cancer with BRCA1 gene defects, with good results.^13^ PARP1 inhibitors can also enhance the efficacy of radiotherapy, alkylating agents, and platinum‐based chemotherapy by inhibiting DNA repair and promoting apoptosis in tumour cells.^13^ Homologous recombination repair deficiency (HRD), as a necessary prerequisite for synthetic lethality, plays a crucial role in killing tumour cells. However, the mutation rate of BRCA1 mutations in most gastrointestinal tumours is actually not high.^24^ Our study found that in patients with GC without BRCA1 mutations, the PARP1 inhibitor OLP combined with AIL inhibiting BRCA1, and they work hand in hand to inhibit HR and BER pathways with powerful anti‐tumour effects.

In summary, the inhibitory effect of AIL on BRCA1 made cancers with normal BRCA1 function sensitive to OLP's PARP inhibition when they are used together. This greatly expands the range of applications of PARPi.

## AUTHOR CONTRIBUTIONS

**Chunming Wang:** Conceptualization (equal); data curation (equal); formal analysis (equal); funding acquisition (equal); investigation (equal); resources (equal); software (equal); supervision (equal); writing – original draft (equal). **Tingzhuang Yi:** Conceptualization (equal); data curation (equal); resources (equal); software (equal); visualization (equal). **Xiangde Li:** Investigation (equal); methodology (equal); resources (equal); software (equal); writing – original draft (equal). **Jiarui Cui:** Investigation (equal); writing – original draft (equal); writing – review and editing (equal). **Biqi Li:** Visualization (equal); writing – original draft (equal). **Yankai Qin:** Investigation (equal); validation (equal); visualization (equal). **Shixiong Tang:** Funding acquisition (equal); investigation (equal). **Jianfeng Zhang:** Data curation (equal); formal analysis (equal); funding acquisition (equal); investigation (equal); supervision (equal).

## FUNDING INFORMATION

The research was supported by The Guangxi Young and Middle‐aged Teachers Basic Ability Promoting Project (grant no. 2022KY0105); The Open Project of Guangxi Key Laboratory of Regenerative Medicine (grant no. 201904); Guangxi Postdoctoral Special Fundings (grant no. 202110); Joint Project on Regional High‐Incidence Diseases Research of Guangxi Natural Science Foundation (grant no. 2022JJA141119).

## CONFLICT OF INTEREST STATEMENT

The authors have no conflict of interest to declare.

## CONSENT FOR PUBLICATION

All authors have agreed to publish this manuscript.

## Supporting information


Figure S1.

Figure S2.

## Data Availability

The datasets from the current study are available from the corresponding author on reasonable request.
