# Supplementary figures and images for: Ailanthone synergizes with PARP1 inhibitor in tumour growth inhibition through crosstalk of DNA repair pathways in gastric cancer
Source: J Cell Mol Med. 2023 Nov 27;28(2):e18033. doi: 10.1111/jcmm.18033 (PMC10826444; doi:10.1111/jcmm.18033)

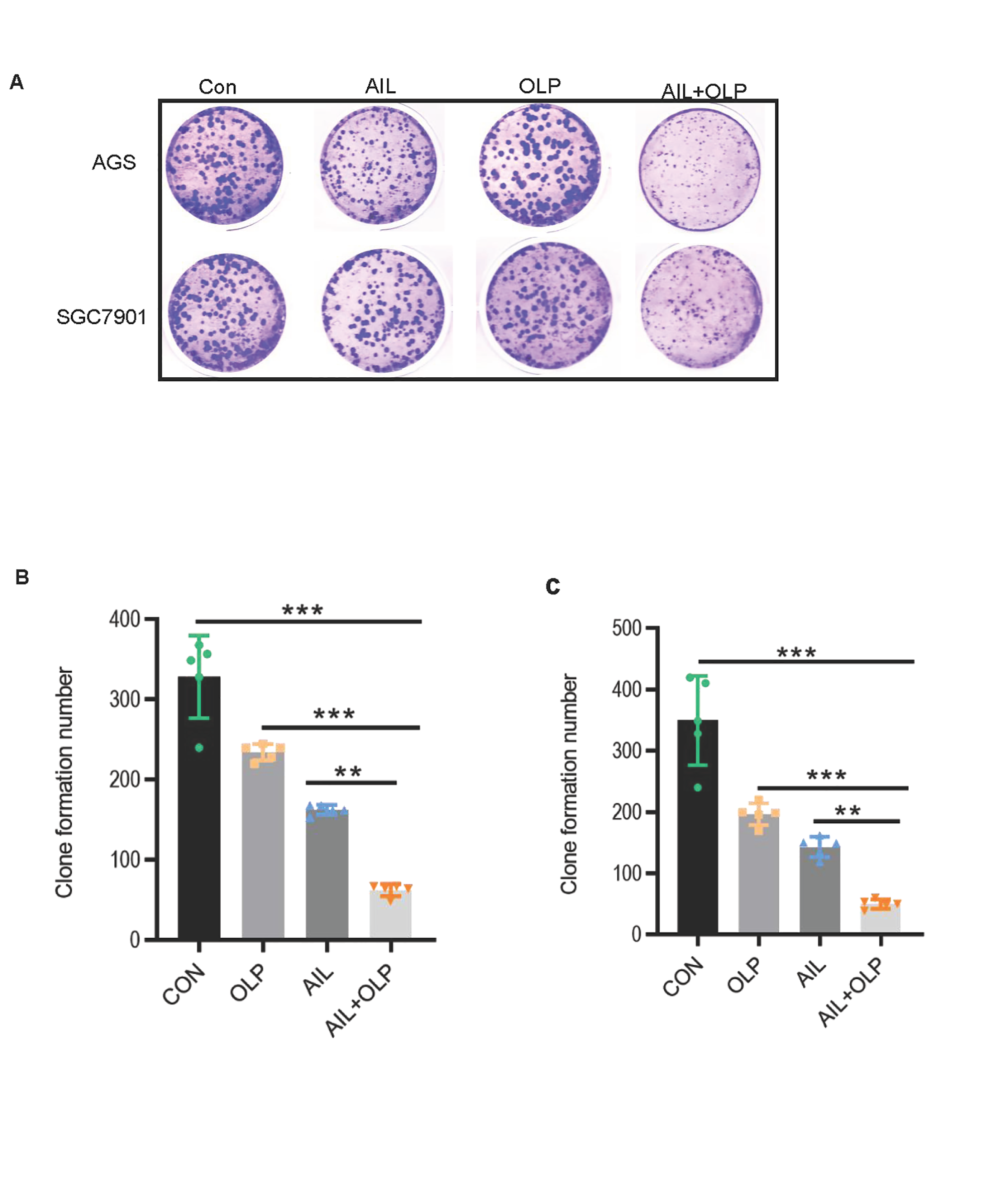

Supplement: Supplementary file 1 — Figure S1. [file JCMM-28-e18033-s001.tiff]

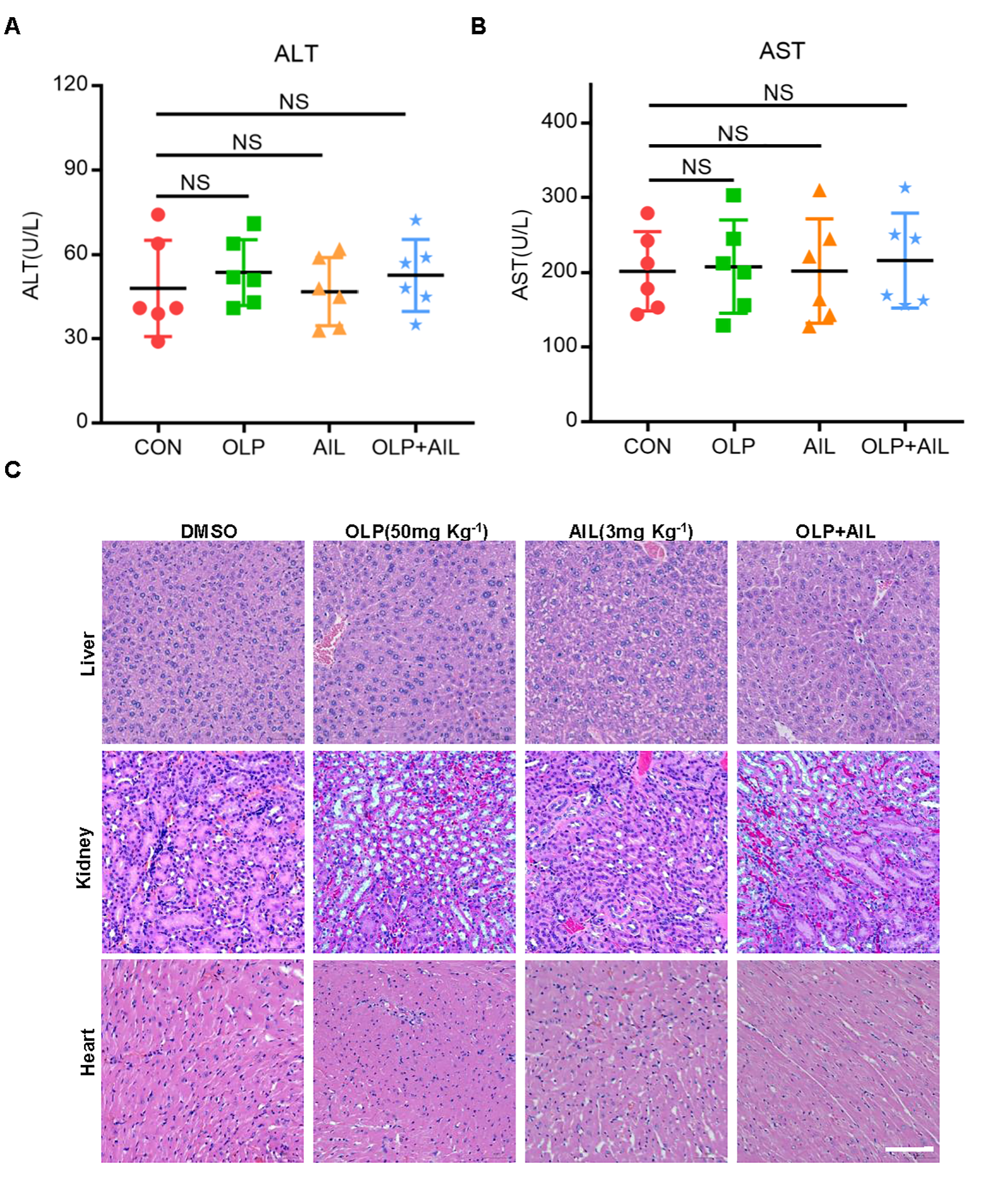

Supplement: Supplementary file 2 — Figure S2. [file JCMM-28-e18033-s002.tiff]
